# Hybrid machine learning approach for landslide prediction, Uttarakhand, India

**DOI:** 10.1038/s41598-022-22814-9

**Published:** 2022-11-22

**Authors:** Poonam Kainthura, Neelam Sharma

**Affiliations:** 1 School of Computer Science, University of Petroleum and Energy Studies, Dehradun, India; 2 Department of Computer Science, Banasthali Vidyapith, Jaipur, Rajasthan India

**Keywords:** Natural hazards, Engineering, Mathematics and computing

## Abstract

Natural disasters always have a damaging effect on our way of life. Landslides  cause serious damage to both human and natural resources around the world. In this paper, the prediction accuracy of five hybrid models for landslide occurrence in the Uttarkashi, Uttarakhand (India) was evaluated and compared. In this approach, the Rough Set theory coupled with five different models namely Bayesian Network (HBNRS), Backpropagation Neural Network (HBPNNRS), Bagging (HBRS), XGBoost (HXGBRS), and Random Forest (HRFRS) were taken into account. The database for the models development was prepared using fifteen conditioning factors that had 373 landslide and 181 non-landslide locations that were then randomly divided into training and testing locations with a ratio of 75%:25%. The appropriateness and predictability of these conditioning factors were assessed using the multi-collinearity test and the least absolute shrinkage and selection operator approach. The accuracy, sensitivity, specificity, precision, and F-Measures, and the area under the curve (AUC)-receiver operating characteristics curve, were used to evaluate and compare the performance of the individual and hybrid created models. The findings indicate that the constructed hybrid model HXGBRS (AUC = 0.937, Precision = 0.946, F1-score = 0.926 and Accuracy = 89.92%) is the most accurate model for predicting landslides when compared to other models (HBPNNRS, HBNRS, HBRS, and HRFRS). Importantly, when the fusion is performed with the rough set method, the prediction capability of each model is improved. Simultaneously, the HXGBRS model proposed shows superior stability and can effectively avoid overfitting. After the core modules were developed, the user-friendly platform was designed as an integrated GIS environment using dynamic maps for effective landslide prediction in large prone areas. Users can predict the probability of landslide occurrence for selected region by changing the values of a conditioning factors. The created approach could be beneficial for predicting the impact of landslides on slopes and tracking landslides along national routes.

## Introduction

Natural disasters are important problems all around the world, and many governments spend a large portion of their annual budget trying to regulate and prevent them^1^. Landslides are widespread in hilly and mountainous places, and they result in significant losses of life and property, having a devastating impact on the socioeconomic situation of a region^2^. Landslides occur when shear pressure in the inclination exceeds shear quality^3^. It has a significant impact on slope modification, particularly in terms of height, steepness, and slope shape^4^. Landslides can be found on all continents and play a significant role in landscape change. They also pose a severe threat to the inhabitants in many locations^5^. Landslides are frequently influenced by both natural and manmade factors^6^. The escalation of human engineering operations in recent years has not only aided in the development of society and the economy but has also contributed to climate instability and the potential increase in the socio-economic value of landslides^7,8^. Therefore, early spatial prediction of landslides is desirable to avoid landslides and, as a result, damage^9^. There were 4862 landslides reported worldwide between 2004 and 2016, resulting in 55,997 deaths^10^. In this aspect, rigorous planning is required to reduce landslide hazard risk, loss, and slope instability mitigation^11^. Developing a landslide mapping model is a fundamental step toward creating catastrophe assessment and mitigation measures in high-risk locations^12–15^. The study of landslide prediction modeling has become a major scientific topic around the world due to its enormous importance^16–18^.

Researchers have devised many ways for identifying landslide-prone areas as well as solutions for reducing the negative effects of landslides^19–26^. Landslide susceptibility mapping (LSM) is one of the most effective approaches for predicting landslide-prone zones in certain areas^27^. In general, future landslides in a given location are expected to occur under comparable conditions as in the past^28^. As a result, a spatial relationship between the elements that influence the occurrence of landslides is necessary to identify and predict future landslide locations^29^.

The terminology and methods used in the field of landslide prediction modeling have evolved over the years, and they now encompass both qualitative (inventory-based and knowledge-driven)^15,28^ and quantitative (data-driven and machine learning) ^30–32^ techniques. However, the best strategy for determining landslide occurrence is still up for debate^33^. Experts utilize qualitative methods to assess landslide vulnerability zones based on their knowledge^34^. Expert knowledge is derived from a combination of field studies and theoretical understanding of physical processes^34^. Knowledge-based models^35,36^, such as the weighted linear combination, rank the relevance of landslide conditioning elements based on expert opinions and experience^37^. Additionally, the qualitative procedures such as the Analytical hierarchy process (AHP)^38^ are based on the opinions of one or more experts^39–41^. The complex non-linear link between landslide and landslide producing causes is recovered in a knowledge-driven model by giving weight to elements based on expert knowledge^42^. Expert knowledge is crucial to the success of the knowledge-driven paradigm^15,43^. These methods, on the other hand, are costly and require a high level of geology and geomorphology knowledge^44^. However, it is difficult to objectively assess or evaluate the quality of the outcomes using these methods^28^.

On the other hand, quantitative approaches^45^ generate numerical estimates, i.e., probabilities of landslide occurrence in any susceptibility zone^46^. Methods such as frequency ratio (FR)^47^, logistic regression (LR)^48^, statistical index (SI)^49^, the weight of evidence (WoE)^50^, evidential belief function (EBF)^51^, information value (IV)^52^, certainty factors (CF)^53^, multivariate regression (MR)^54^, are based on strong mathematical rules, regardless of individual decision^55^. The strategies listed above have been utilized by researchers all across the world to predict landslides^37,41,56–58^. These data-driven or statistical models are built on simple principles that can outperform simple univariate and multivariate linear tasks^10^. Quantitative techniques for landslide prediction modeling have risen in prominence over the last two decades^15^. Even though various methods for assessing landslide dangers have been developed, no method has been acknowledged as the standard technique for analysis and prediction^59^. Conventional statistical approaches do not perform well for complex and high-dimensional nonlinear issues^60^. As a result, the data nature is likely to change in many circumstances, lowering the model accuracy^61^.

Machine Learning approaches are the most effective at handling complicated and nonlinear high-dimensional^62^ data sets in landslide research. Tree inductions^63^, probabilistic approaches^21,22^, Artificial Neural Network (ANN)^23^, and Support Vector Machine (SVM)^19^ are among the methods used. Machine learning together with artificial intelligence^64,65^ algorithms has shown to be an effective and promising tool in many geotechnical applications^66^. In landslide forecasting, machine learning methods are being utilized to improve model accuracy and, in particular, the flexibility of such models to handle a wide range of conditioning factors^7,67^. Landslide mapping has vastly improved in the recent decade, largely in major part to machine learning techniques^68,69^. These models, however, still include some shortcomings, and these shortcomings are impacting the prediction performance of single models^70^. When training data is limited, for example, there is a risk of underfitting, which can lead to erroneous model development when employed alone^71^. Ensemble approaches like Bagging^72^, Boosting (AdaBoost^73^, Gradient Boosting Machine (GBM)^24^, Extreme Gradient Boosting (XGBoost)^74^), and Random Forest^75^ were developed in the field of machine learning to help solve this challenge. Ensemble techniques are a type of machine learning methodology that integrates numerous base models to create a single best-fit predictive model. By gaining a better knowledge of the data and rules from multiple models, an ensemble can reduce variance and bias^70^. Ensemble methods aid in the reduction of over-fitting issues in models^76^. It also works well with data with a range of dimensions. Furthermore, the computation performance is unaffected by missing values in the dataset^77^. Additionally, it can also deal with problems in unbalanced data and error reduction^70^. Compared to other ensemble models, the XGBoost technique provides many advantages^78^. Outliers have a negligible effect. There is no need to scale or normalize the data, and it can even manage missing values. The training time is greatly reduced by parallelizing the entire boosting procedure^79^.

Model ensembles, on the contrary hand, are not always preferable^80^. Bagging has one limitation: instead of reporting specific values for the classification or regression model, it focuses its final prediction on the mean predictions from the subgroup trees^81^. Overfitting is possible in boosting if parameters are not tuned properly^71^. Overall, it is observed that the capability of the individual model has some limitations^82^.

Therefore, hybrid machine learning algorithms have recently generated promising results in landslide prediction modeling^23,83,84^. The base model prediction performance is improved by using a hybrid technique^10^. Moreover, the hybrid models higher performance in predicting landslide revealed that the landslide modeling may be improved by factor optimization^85^. Many studies have demonstrated the value of a hybrid strategy in landslide situations all over the world^10,82,83,86^. Due to regional geological and geomorphological causes, slope unsteadiness, including landslides, is a common problem in Uttarkashi, Uttarakhand^87–89^. According to NASA statistics, 958 landslides killed 6779 persons in India between 2008 and 2015, with Uttarakhand leading the way with 5,226 deaths^90^. Various scholars discovered the reason for landslides in the Uttarkashi region after extensive research^68,91–93^. In the research area, the progression of road construction and changes in land-use patterns have been recognized as the major causes of landslides^94^. Several well-known and well-tested machine learning algorithms, such as SVM, Naïve Bayes, Logistic Regression, Bayesian Network, BPNN, and Random forest were used in this study because they produce great results in the situation of landslide assessment^68,93^. The ability to forecast using various combinations of machine learning technologies is always improving^83^. To select the optimal model, we investigated five hybrid models namely XGBoost-Rough Set (XGBRS), Backpropagation Neural Network-Rough Set (BPNNRS), Bayesian Network-Rough Set (BNRS), Bagging-Rough Set (BRS), and Random Forest-Rough Set (RFRS) in the landslide prediction modeling of the Uttarkashi district, Uttarakhand, India. Additionally, as a result of geological and geomorphological changes induced by climate change, landslide risk may appear in non-landslide areas of study area^95^. Therefore, utilizing GIS platforms such as Openstreet maps^96^, the hybrid model with optimal prediction capabilities is used to predict landslide event based on changing conditions. By evaluating and comparing models using multiple statistical indicators^97^, the best prediction model will be found. The primary distinction between this study and earlier ones is that it is the first time that advanced XGBoost ensemble optimization techniques combined with Rough Set are used to investigate the possibility of a more accurate landslide model in the Indian Himalayan region. The main goal of the research is to find an appropriate machine learning algorithm for identifying possible landslide regions in the Uttarkashi region.

**Figure 1:**
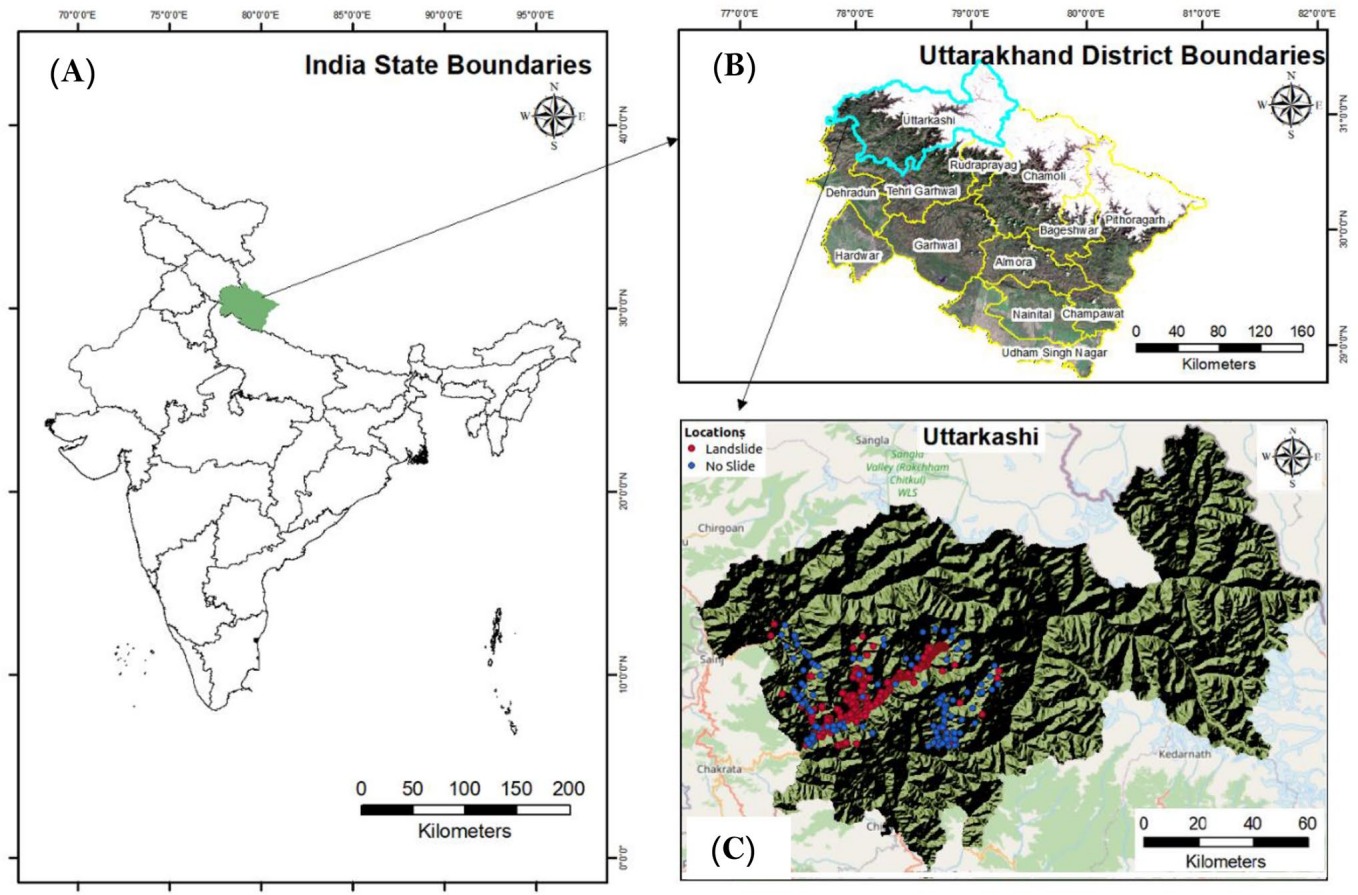
Location map of the study area (**A**) state boundaries of India and study area (Uttarakhand state) (**B**) district boundaries of Uttarakhand state with Uttarkashi district highlighted and (**C**) showing the landslide (red) and non-landslide(blue) locations of Uttarkashi district.

Thus, the primary objective of this investigation is to assess the impact of the following on the accuracy of landslide prediction modelling: (1) pre-processed landslide data inventory; (2) landslide-conditioning factors (LASSO and Multicollinearity); (3) prediction techniques; (4) selective validation methods and statistical measures; and (5) visualising the outcome using interactive maps. A case study of the Uttarkashi district (Fig. 1) is established based on the use of 554 landslide/non-landslide locations and 15 conditioning factors to support the proposed methods.

### Study area

The study area as shown in Fig. 1, is in the Uttarkashi district between 30.7268° N latitude and 78.4354° E longitude, with an area of approximately 8016 km^2^. The map of the study area is generated using ArcGIS v10.0 software. The study area is famous for pilgrimage that is located in the NW direction of Uttarakhand state. During the monsoon season from July to September the study area receives heavy rainfall and from January to March months it receives moderate rainfall^98^. The average rainfall recorded is 1902 mm. The elevation varies from 920 to 3830 m and the average altitude calculated is 1158 m. Failure of slopes in these regions is very frequent during a rainstorm. The state northern portions are characterized by the Greater Himalaya highlands, which are dominated by towering Himalayan peaks and glaciers, with lush vegetation covering the lower foothills. The Indo-Gangetic plain, with a maximum elevation of fewer than 1200 m, is home to the southernmost districts. Uttarakhand is drained by the Ganges river system, with the Ganga, Yamuna, and Kali being the main glacially fed rivers that originate in the province Greater Himalayan areas. Every year, massive landslides occur in the study area as a result of heavy rainfall, steep slopes, and road cutting. Academicians are interested in understanding the landslide activities that occur there and proposing a solution in the field of landslide modeling for those ongoing environmental processes because of the immense diversity and complexity of the environment. Landslides occur regularly throughout the study area, inflicting damage to roads, buildings, and economic infrastructure. Ongoing tectonic activity, extreme rains, and water seepage into the slopes, shear planes, weak lithology, and man-made activities are all contributing to the instability of these Himalayan slopes. The study area was determined by the availability of landslide occurrence causes as well as exact slide locational data. Furthermore, the research field has a substantial challenge that must be addressed using scientific methods.

## Methodology

**Figure 2:**
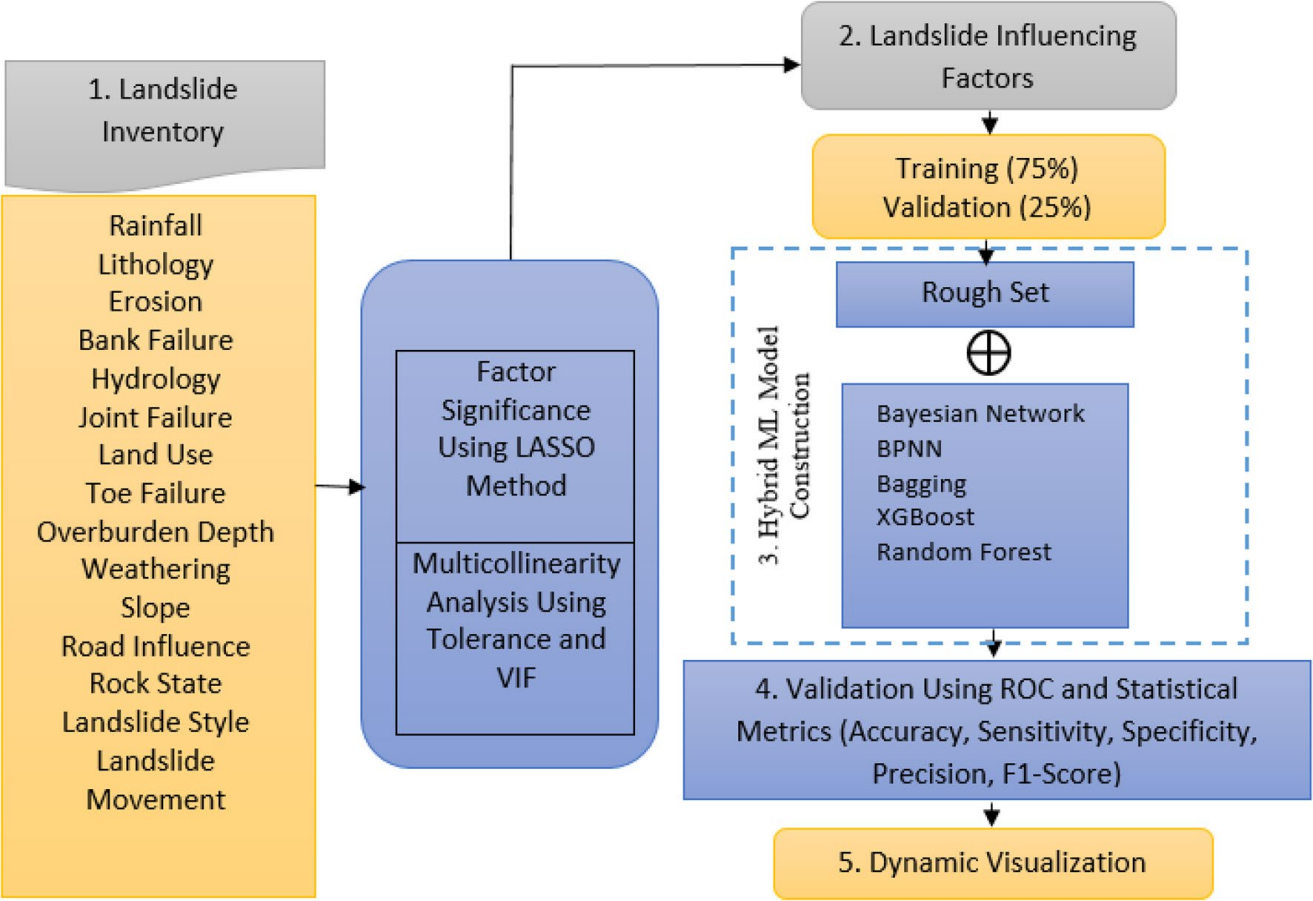
Methodological flowchart showing different steps for the construction of hybrid machine learning models for the prediction of landslide events supported by LASSO method.

To prepare the landslide prediction model of the study area, the research was divided into five phases: The workflow of the methodology is shown in Fig. 2.

### Data collection and pre-processing

The creation of a landslide inventory is required for landslide forecasting. Landslide databases record the locations and conditions of landslides that have migrated in the past, but the mechanism(s) that caused them to move is rarely indicated. This can provide historical and current landslide sites, occurrence periods, landslide types, frequency and severity of occurrence, magnitude and extent, failure mechanisms, causal-related factors, damages, and consequences. This comprises reports based on field studies prepared by the Geological Survey of India (GSI, https://www.gsi.gov.in/webcenter/portal/OCBIS/pageReports/pageGsiReports?_adf.ctrl-state=1gq1usi84_5&_afrLoop=935857055668031#!). Past landslide spots in Uttarkashi District were used as markers (Latitude, Longitude). The coordinates and other details are taken from publicly available landslide records. A total of 554 landslides with precise characteristics were recorded in the study area. Because the dataset contains missing values, a data preparation process was used to handle the data. This phase will aid in the model prediction capability improvement. The quality of the datasets and the models utilized determine the final data quality. Previous research has utilized a variety of ways to clean the data. Statistical approaches including mean and median computations, Multivariate Imputation by Chained Equation (MICE)^99^, Imputation Using Deep Learning^100^, K-Nearest Neighbor^101^, and others are included. In this study, a popular method K-Nearest Neighbor model (KNN) was utilized to the fill missing values to build a high-quality dataset for training. The KNN model, based on the Euclidian distance formula mentioned in Eq. (1), is employed in this work to fill missing values using the KNN Imputer function.

1 $$Distance\left( {X_{i} ,Y_{i} } \right) = \sqrt {\mathop \sum \limits_{i = 1}^{n} \left( {X_{i} - Y_{i} } \right)^{2} }$$

where $X_{i}$ and $Y_{i}$ represent observed and actual data values, respectively, and "n" represents the total number of instances.

**Figure 3:**
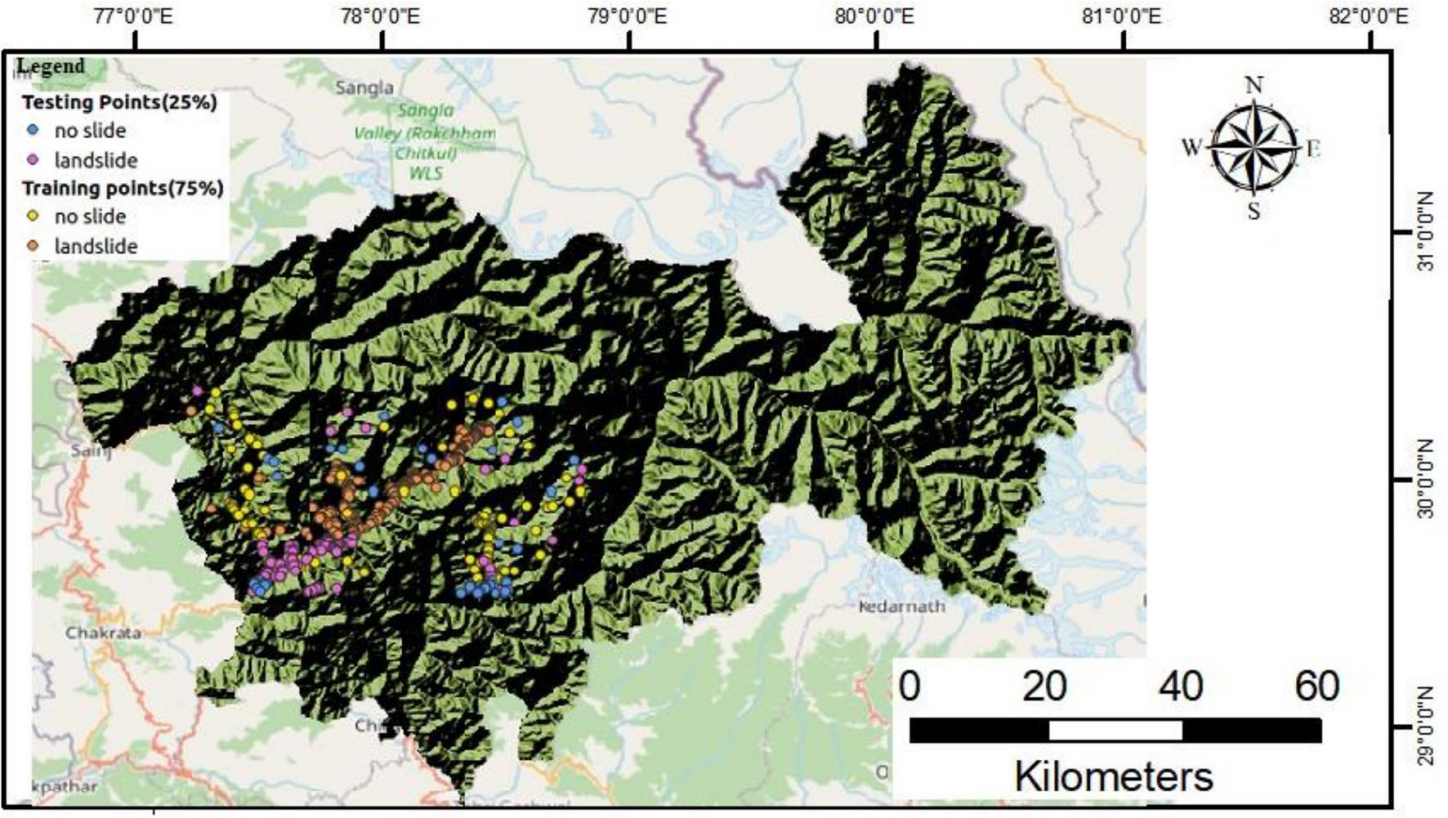
Partition of the landslide and non-landslide locations into training and testing data points with in ratio of 75:25.

As a result, the landslide inventory prepared and listed in Table 1, is valuable for determining the spatial distribution of present landslides as well as the potential for future landslides. The total inventory of landslides was then randomly divided into two data sets with 279 landslides and 136 non landslides for training (75%) and 94 landslides and 45 non landslides for testing (25%). Figure 3 displays the training and testing split of landslide locations. Total of fifteen landslide conditioning factors (LCFs) was used in this study for landslide prediction modeling in the Uttarkashi district. The description of each landslide conditioning factor is given below:

**Table 1:** Landslide Inventory sample of the study area (Uttarkashi district) obtained based on field investigation that is publicly available on GSI landslide reports ^19–21^.

Parameters	Values			
(Latitude, Longitude)	30° 48′ 36.9" 78° 13′ 10.7"	30° 48′ 35.5", 78° 13′ 8.5"	30° 48′ 40" 78° 13′ 6.2"	30° 48′ 50.9 78° 13′ 18.9"
Rock State	Massive	Sheared	Fractured	Jointed
Hydrology	Dry	Wet	Damp	Dry
Weathering	Low	Moderate	High	low
Overburden Depth(m)	> 5	0–1	2–5	0–1
Erosion	No	No	yes	No
Rainfall	Yes	No	No	Yes
Road Influence	No	No	No	No
Slope	Steep	Moderate	Gentle	Steep
Joint failure	No	No	No	No
River Bank Failure	No	No	No	No
Toe failure	No	No	Yes	Yes
Land Use	Dense	Agriculture	Sparse	Moderately Vegetated
Lithology	Slates	Gneiss	Quartzite	Slates
Landslide Movement	Rotational	Translational	Debris	Debris
Landslide Style	Single	Complex	Multiple	Complex
Landslide Prediction	Yes	No	Yes	No

#### Erosion

Erosion is characterized as a physical process with significant global variation in intensity and frequency that is influenced by a variety of social, economic, and political issues in addition to environmental factors. The importance of erosion (channel incision, lateral channel migration, and slope erosion owing to agricultural runoff) as a landslide trigger cannot be underestimated^102^. Massive erosion is a type of erosion that occurs when a large amount of soil or rock mass, or a combination of both, is pushed down a slope by gravity. In fact, this erosion happens when the weight of the material exerts a force greater than the resistance force imposed by soil shear force. Massive erosion in the study area is typically associated with natural erosion, but human activities, such as mine excavation, road construction, and forest vegetation degradation, exacerbate this (deforesting). Massive erosion contribute to landslides, and underground erosion is one of the mechanisms that contributes to soil mass instability. The study area Uttarkashi is prone to landslides due to discussed erosional activities^103^.

#### Lithology

Lithology describes the physical characteristics of a rock unit, such as color, texture, size, and composition. Lithology is one of the most essential parts of landslide study since different lithological units have different geological strength indices, permeability, and susceptibility to failure. As a result, lithology is commonly considered one of the most critical landslide conditioning factors^28^.

#### Bank failure

There are various small and major rivers in the research area. The largest and most revered of these rivers are the Bhagirathi and Yamuna. Cutting the river banks in these locations puts people's lives in jeopardy. Construction, weight on the riverbank, vegetation, tectonic activity, water saturation, and other conditioning factors can all contribute to river cut failure. As a result of these conditioning factors, stress develops along riverbanks, weakening the soil strength and increasing the risk of landslides^104^.

#### Hydrology

Hydrology is the study of the water cycle, water resources, and watershed sustainability on Earth and other worlds, as well as water transportation, distribution, and management. Anthropogenic activities are continually disturbing the Himalayan environment's natural system, with apparent results in the hydrology of streams and springs^105^. The variation of water discharge with the seasons and the increasing amount of sediment load in streams are two of the most important challenges in the study region. The majority of the locations along the stream that runs through the slide zone are dry to damp.

#### Rainfall

In the studied region, the average annual rainfall is 1902 mm. The most common and widespread destructive landslides are caused by prolonged or intense rainfall. Extreme rainfall events in landslide-prone areas can be disastrous, causing property, infrastructure, and human life to be lost. At several spatiotemporal scales, research on the rainfall characteristics known to produce landslides has been done, and it frequently relies on local knowledge of landslides and rainfall. Rainfall has increased the Himalayan mountains fragility, resulting in a rise in the number of landslides in the region. The majority of rain-caused landslides are shallow, small, and move swiftly. Many rainfall-induced landslides become debris flows as they descend steep slopes, especially those that enter stream systems, where they may mix with extra water and sediment^106^.

#### Joint failure

Landslide distribution is influenced by faults/fractures and other structural lineaments. Local stress fields are assumed to be reflected by different-trend faults/fractures, which have an unequal influence on landslides. As we all know, joints govern the slope and are associated to rockfall. A cross joint positioned NE-SW and perpendicular to the fold axis, a longitudinal, sub-vertical joint oriented WNW-ESE to NW–SE, and a shear joint oriented diagonally from NNW-SSE to ENE-WSW are among the joints in the research area. These joints can sometimes lose control due to the fractured and massive rock, resulting in a landslide^107^. As a result, joint failures are viewed as a key triggering factor in the research.

#### Land use

Land cover is the physical material that covers the earth's surface. Examples include grass, asphalt, trees, bare ground, water, and other land covers. Land cover/land use has an impact on landslide occurrences because the roots of trees in the forest region play an important role as anchors in retaining the stability of soils and rocks on the slope. Natural vegetation cover on a slope (Landcover), as well as anthropogenic activities such as agriculture, plantation, and large excavation for roads and other civic constructions (Landuse), have a direct impact on hillslope stability. Extensive Cutting is locations along the road and river where extensive toe removal is found in the research region, either as a result of anthropogenic or natural conditions. Wasteland refers to both cultivable lands that have been abandoned and areas where materials (trash) have been deposited. Landslides are associated with barren, massive slope cuttings, and thinly vegetated places. Understanding how different land coverings affect landslides is increasingly required for developing landslide prediction models^107^.

#### Toe failure

In the investigated area, slope toe cutting is sometimes manmade and most of the time natural. When the toe of a slope is removed, the friction to movement is reduced. Frequent rains, on the other hand, eroded the toe of the slope, deforming the hill. Furthermore, building or widening roads without properly understanding the nature of the slope might lead to toe collapse, which can increase landslide occurrences^108^.

#### Overburden depth

Overburden refers to the material (in-situ or transported) that exist on the surface of young rock formation, such as soil and weathered mass^109^. The thickness of the overburden is determined by a variety of characteristics, including terrain shape, slope forming material, weathering rate, slope degree, and so on. Shallow translational debris landslides have been linked to the depth of the overburden to the bedrock. It is also affected by slope and erosion. The overburden depth could be beneficial for recognizing landslide-prone regions and constructing prediction models because the research area is prone to erosion and includes steep slopes. In this study, the overburden depth factor spans from 0–1 m to > 5 m.

#### Weathering

The weathering and landslides in this mountainous tract in India's northern region have been influenced by the meteorological conditions^110^ in the Uttarakhand region. Landslides are widespread in the study area during the monsoon season, and their severity is determined by the thickness of the loose, unconsolidated soil created by weathering. Strong weathering and tectonic activity can be seen near the thrust and fault in the research region. Other conditioning factors speeding up the weathering process and causing landslides in the region include deforestation, land-use practices, and soil erosion. The power of weathering might be low, moderate, or high, depending on the geology and climatic circumstances. When the power is really high, landslides are more likely.

#### Slope

The steepest direction is always used to calculate the angle of all slopes. When the slope angle of a viscous material increases, the shear strength of the material generally falls^111^. There are several types of slope angles, ranging from 0 to > 35 degrees. The range of slopes is divided into three categories: gentle (0–8.5), moderate (8.5–35), and steep (> 35).

#### Road influence

The anthropogenic component of road construction (cutting and excavation) has a major impact on natural slope morphology^112^. Road construction activities degrade the mountain's structural stability along the corridor, increasing the risk of landslides. Where roads and highways cross steep terrain, maintaining the stability of road cuts and road terracing is vital. Furthermore, water must be diverted away from slopes so that the road does not block water flow and the groundwater level beneath the road's surface is not too shallow. Natural landslides may occur, causing road instability, road cuttings, and earthworks. Road cuttings and earthworks that are unstable can cause the road to disintegrate, and become dangerous, and inaccessible.

#### Rock state

The landslide research area has phyllite, quartzite, argillaceous, dolomite, and other rocks. The existence of structural discontinuities in the stones, such as faults, joints, bedding, and thrusts to slope inclination and direction, has a significant impact on slope stability. According to the recorded data, the rock status for the specified study location is classed as massive, sheared, fractured, and jointed. As a result, it could be involved in both the initial rockslide and the secondary rock avalanche. Bonding determines the cohesiveness and internal friction angle of a rock's shear strength. Mechanical fractures in rocks cause displacement discontinuities across surfaces or narrow zones, which are known as fractures^113^.

#### Landslide movement

A landslide is defined as the movement of a rigid body of earth or land along a shear surface. A slide is defined as a series of mass motions that remove the slide mass from more secure earth material in weak zones. Inside the research region, there are a variety of landslide types, including translational, rotational, or combination rock-debris slides, as well as ordinary debris slides. A rotational occurs when the surface is curved and concave to the sky. Moreover, the upper side of the slide is inclined in the backward direction towards the original slope and the lower surface leaves the slope. Whereas translational slide moves in a downward and outward direction inclined on upper planar surface developing dangerous situation by flowing debris on steep ground^6^.

#### Landslide style

Various landslide activity styles have been seen in the Uttarakhand study region. Single, complex, and multiple styles were the most commonly recorded. A single landslide is the flow of displaced material in a single direction. At least two forms of movement (falling, toppling, sliding, spreading, and flowing) occur in a complex landslide. Multiple landslides are characterized by the emergence of the same type of movement multiple times^114^.

### Selection of landslide-influencing factors

Landslides are a very complicated geo-environmental event that can be caused by a number of different factors. However, it is also true that not all factors have an equal influence on the initiation of a landslide in any given location. As a result, determining appropriate and accountable factors for the occurrence of a landslide is an important task that demands special consideration in landslide prediction modeling. As a result, careful evaluation of landslide conditioning factor (LCF) influence and removal of the less significant ones from the model is critical. LASSO and Multicollinearity tests are two effective ways to determine the influencing elements for this purpose.

### Independent test of causative factors

The least absolute shrinkage and selection operator (LASSO)^115^ is a widely used and recommended technique used to test the capability of predicting factors^116^. When selecting factors, a larger value implies that the component is more important in the occurrence of a landslide. This is a flexible technique for identifying and regularising features. The LASSO improves model interpretability and avoids overfitting by removing extraneous factors that are unrelated to the response factors. Shrinkage is used in the Lasso regression model. The data values are shrunk towards a center point with this technique, which is comparable to the mean approach. Mathematically, the LASSO method can be expressed as:

2 $$\mathop \sum \limits_{i = 1}^{n} \left( {y_{i} - \mathop \sum \limits_{j} x_{ij} \beta_{j} } \right)^{2} + { }\lambda \mathop \sum \limits_{j = 1}^{p} \left| {\beta_{j} } \right|{ }$$

Which is representing the same as minimizing the sum of squares error that is the first component of formula shown in Eq. (2) with constraint component $\lambda \mathop \sum \limits_{j = 1}^{p} \left| {\beta_{j} } \right|$ dependent on the value of $\lambda$ and $\beta_{j}$. The value of $\beta_{j}$ can be shrunk to exactly zero.

Where, λ is the size of the shrinkage. If λ = 0, it signifies that all features are considered, and it is now the same as linear regression. On the other hand, if λ = ∞ it signifies that no feature is used; as the number grows larger, more features are discarded, and feature selection gets more precise. Additionally, the value of rises as the bias increases. The value of falls as the variation grows.

### Multicollinearity test of causative factors

Using several machine learning methods, the study incorporated fifteen landslide conditioning factors to measure landslide prediction. When two or more predictors are associated, the standard error of the coefficients increases. This is known as multicollinearity ^117^. In landslide prediction modeling, checking the multicollinearity of multiple conditioning factors is critical. This analysis can help with attribute selection as well as understanding which conditioning factor have the most impact on the target features to predict future landslides. The tolerance and variance inflation factor (VIF) was used to determine the proper assessment of multicollinearity among landslide causative parameters. The tolerance and VIF were calculated using Eq. 3.

3 $${\text{VIF}}_{{\text{i}}} = \frac{1}{{1 - R_{i}^{2} }} = { }\frac{1}{{{\text{Tolerance}}}}$$

where *R* is the coefficient of regression of independent conditioning factor “i “*.* When the TOL number is > 0.2, there is no evidence of multicollinearity; on the other hand, a TOL value less than 0.1 suggests significant multicollinearity. The presence of strong multicollinearity among the independent conditioning factor is indicated by a value of greater than 10 in VIF.

### Methods for landslide prediction modeling

#### Bayesian network

A Bayesian Network^70^ is a statistical classifier that shows a probabilistic link between several conditioning factors. BN is a powerful technique for modeling a complicated causal network system. BN preserves the joint probability among causal components in the form of an acyclic directed graph (DAG)^118^. When multiple causal elements are coupled, they might have a lot of power. Each node in the graph keeps a conditional probability table to illustrate cause-and-effect interactions (CPT). The BN model can learn cause-and-effect networks quickly and can tolerate partial datasets. The CPT computation for each causal element of a landslide is expressed as Eq. (4).

4 $$P\left( {X_{i} {|}Y} \right) = P\left( {Y{|}X_{i} } \right){*}\frac{{P\left( {X_{i} } \right)}}{P\left( X \right)}$$

The combined probability of predicting landslide based on numerous parameters is expressed as Eq. (5)

5 $$P\left( {X_{i} } \right) = \mathop \prod \limits_{i = 1}^{n} P(X_{i} |predecessor\left( {Y_{i} } \right))$$

where $X_{i}$ the number of cases, $Y_{i} { }$ is a set of causative factors, $P(X_{i} |predecessor\left( {Y_{i} } \right)$ denotes that $X_{i}$ is the cause of $Y_{i}$, and $P\left( {X_{i} } \right)$ is the probability of all possible $X_{i}$ values.

BN is thought to be a potential strategy for predicting landslides^119^. It is, however, only seldom used to predict landslides. Landslide stability is influenced by a variety of elements, which are divided into internal and external influence components. Landform, stratum structure, rock and soil characteristics, and hydrological elements are among the internal components.

#### Artificial neural network

The artificial neural networks (ANN)^120^ is a form of machine learning algorithm that learns by finding valuable patterns from data on its own. Artificial neural networks, like the human brain, have neurons in multiple layers that are coupled to one another. Three layers make up Artificial Neural Networks: (1) Input Layer, (2) Hidden Layers, and (3) Output Layer^30^. In prediction and regression applications, the back-propagation neural network (BPNN)^121^ learning technique is the most often employed ANN model. Because of the nature of the backpropagation learning mechanism, backpropagation neural networks can be utilized to tackle issues in a wide range of domains. This method is employed in any sector where neural networks are used to solve problems involving a set of inputs and a set of output targets^121^. The backpropagation algorithm's training is divided into three parts. The training input is fed forward in the first stage. The calculation and backpropagation of linked mistakes is the second stage. The sigmoid function is used to calculate mistakes, and Eq. (6) is the mathematical expression utilized to calculate the error.

6 $${\text{Error}}_{{{\text{BP}}}} = {\text{Layer}}_{{{\text{Outputs}}}} \left( {1 - {\text{Layer}}_{{{\text{Outputs}}}} } \right)\left( {{\text{Output}}_{{{\text{Target}}}} - {\text{Layer}}_{{{\text{Outputs}}}} } \right)$$

where

${\text{Error}}_{{{\text{BP}}}}$ represents the calculated back propagated error,

${\text{Layer}}_{{{\text{Outputs}}}}$ is the actual output of ‘Layer’, ${\text{Output}}_{{{\text{Target}}}}$ is known target value of the training tuple.

Using Eq. (7), modify the relevant weight until it has a minimum error in the third stage.

7 $${\text{W}}_{{{\text{i}}j_{new} }} = {\text{W}}_{{ij_{old} }} + \left( {{\text{Error}}_{{{\text{BP}}}} {\text{*Layer}}_{{{\text{Outputs}}}} } \right)$$

The updated weight is ${\text{W}}_{{{\text{i}}j_{{{\text{new}}}} }}$ while the original or previous weight is ${\text{W}}_{{ij_{old} }}$. The weight is adjusted until the smallest variance is reached if the output does not match the target. Finally, BPNN^68^ may be used to forecast the probability of a landslide.

#### Bagging

A Bagging^122^ is a meta-estimator that fits base classifiers to random subsets of the original dataset, then combines their predictions (through voting or averaging) to get a final prediction^73^. Each base classifier is trained in parallel with a training set formed by replacing 'n' samples (or data) from the original training dataset with new data at random. Where 'n' is the original training set's size. The training sets for each base classifier are distinct from one another. Many of the original data points may be reproduced in the final training set, while others may be eliminated. By averaging or voting, the Bagging approach lowers overfitting; nevertheless, this increases bias, which is countered by the loss in invariance. For a given training set $D = \left\{ {\left( {x_{1} , y_{1} } \right), \ldots , \left( {x_{n} , y_{n} } \right)} \right\}$, sample T sets of n elements from dataset $D_{i} = \left( {D_{1} , D_{2} , \ldots , D_{T} } \right)$ are chosen using the replacement procedure. On each $D_{i}$, ($i = 1, \ldots , T)$ train a landslide model and acquire a sequence of T outputs $f_{1} \left( x \right), \ldots ,f_{T} \left( x \right)$. The final aggregate classifier for the majority of votes is expressed in Eq. (8):

8 $$\overline{f}\left( X \right) = {\text{sign }}\left( {\mathop \sum \limits_{i = 1}^{T} {\text{sign }}\left( {f_{i} \left( X \right)} \right)} \right)$$

#### XGBoost

Boosting^122^ is an ensemble modeling strategy that aims to create a strong classifier out of a large number of weak ones. It's done by putting together weak models to create a strong model. To begin, a model is created using the training data. The second model is then created, which attempts to correct the faults in the first model. This approach is repeated until either the entire training data set is properly predicted or the maximum number of models has been added. Gradient Boosting is a boosting approach in which a new predictor is built each iteration to fit the preceding predictor's pseudo-residuals^26^. Various studies show XGBoost is the better model in comparison to base models^123^. XGBoost^124^ stands for Extreme Gradient Boosting and is a special implementation of Gradient Boosting. XGBoost employs second-order gradients and improved regularisation to get more accurate approximations.

The objective function of XGBoost is the total of all loss functions applied to all predictions, as well as a regularisation function for all predictors (j trees) is shown in Eq. (9).

9 $$obj\left( \theta \right) = \mathop \sum \limits_{i = 1}^{n} l\left( {y_{i} - \widehat{{y_{i} }}} \right){ } + { }\mathop \sum \limits_{j = 1}^{J} \Omega \left( {f_{j} } \right){ }$$

The first component of Eq. (9) represents the training loss that measures how well the model fit on training data and the second component is regularization which measures the complexity of trees. Optimizing training loss encourages predictive models and the same for regularization encourages simple models.

Where the first term denotes the loss function, the second term denotes the regularisation function, and $f_{j}$ denotes a prediction from the j^th^ tree. The final prediction will be achieved through Eq. (10).

10 $$\widehat{{y_{i} }} = \mathop \sum \limits_{t = 1}^{m} f_{t} \left( {x_{i} } \right){ }$$

where $f_{t} \in$Ƒ,${\text{ where}}$Ƒ$\; = { }\left\{ {f_{1} ,{ }f_{2} ,{ }f_{3} ,f_{4} , \ldots f_{m} \} } \right.$ is a set of base learners and $x_{i}$ is representing the feature vector of *i*^th^ data point.

#### Random forest

The Random Forest (RF) model^81^ is a data mining system that classifies large amounts of data accurately using an ensemble of decision trees. Decision trees are predictive models that use a collection of binary rules to select a target class^125^. A set of predictor factors, as well as the class to be predicted, are included in the data used to train the model. The RF model is made up of an ensemble learning approach that connects various landslide decision trees to estimate landslide possibility in a specific area spatially^75^. The RF model divides each node based on the best split in a subset of factors chosen at random by the node. As a result, the smaller the number, the better the split for the node in landslide prediction modeling. For landslide prediction mapping, each node of a normal tree can be split using the ideal split for all landslide prediction parameters. A decision tree built from a specific data set shows anomalies in many of the training data set's branches. One of the most common causes of these anomalies is over-fitting^71^, which can be mitigated utilizing random forest approaches. These statistical techniques are used to classify a set of data. Based on the majority of votes obtained by each model, the final prediction is accepted.

Let $M_{1} , M_{2} , M_{3} , \ldots , M_{i}$ denotes a collection of classifiers used to build a composite model $M^{*}$ on a given dataset $D_{{st_{i} }}$, where *i* denotes the total number of trees used to build a random forest.

$D_{{st_{1} }}$, $D_{2}$ …..$D_{{st_{i} }}$ denotes the various training sets, and n denotes the total number of prediction classes for the models $M_{1} , M_{2} , M_{3} , \ldots , M_{i}$.

The Eq. (11) expresses the entropy or information of the data set $D_{st}$ as:

11 $$Entropy\left( {dataset} \right) = - \mathop \sum \limits_{i = 1}^{n} \left( {\frac{{\left| {D_{{st_{i} }} } \right|}}{{\left| {D_{st} } \right|}}} \right)log_{2} \left( {\frac{{\left| {D_{{st_{i} }} } \right|}}{{\left| {D_{st} } \right|}}} \right)$$

The Eq. (12) aids in obtaining the expected information for a single attribute

12 $$Entropy\left( {attribute} \right) = - \mathop \sum \limits_{i = 1}^{m} \left( {\frac{{\left| {D_{{st_{k} }} } \right|}}{{\left| {D_{st} } \right|}}} \right){*}info\left( {D_{{st_{k} }} } \right)$$

where *k* denotes the number of attributes in total.

The Eq. (13) computes the information gain for each attribute and chooses the information gain with the highest value.

13 $$inf^{Gn} \left( {Attr_{k} } \right) = Entropy\left( {D_{st} } \right) - Entropy\left( {Attr_{k} } \right)$$

where $inf^{Gn} \left( {Attr_{k} } \right)$ denotes the information gain computed for each attribute $Attr_{k}$. This method, however, is biased toward tests with multiple outcomes and prefers to select attributes with a large number of values. The Gain Ratio method addresses the Information Gain method's limitation by normalizing the results using Eq. (14):

14 $$splitinfo = - \mathop \sum \limits_{k = 1}^{m} \left( {\frac{{\left| {D_{{st_{k} }} } \right|}}{{\left| {D_{st} } \right|}}} \right)log_{2} \left( {\frac{{\left| {D_{{st_{k} }} } \right|}}{{\left| {D_{st} } \right|}}} \right)$$

#### Rough set

The rough sets^126^ are used in classification to uncover structural links in noisy and imprecise data. Rough sets naively fit the data before computing membership function values^71^. The preliminary set's categorization is based on the establishment of equivalence classes using the available training data. An equivalence class is made up of tuples that are indistinguishable. There are two parts to the supplied class C: a lower approximation and a higher approximation^127^. The lower approximation of C is made up of all tuples that are based on attribute information and are certain to belong to class C without ambiguity. The upper approximation of C is made up of all tuples that are based on attribute knowledge and cannot be defined as not belonging to class C. Each class has its own set of decision rules, which are displayed in a table.

The lower and upper approximations are expressed in Eqs. (15) and (16):

Upper Approximation:

15 $$\overline{R}X = \cup { }\left\{ {Y{ } \in { }U/R:Y \cap X \ne \emptyset } \right\}$$

Lower Approximation:

16 $$\underline {R} X = \cup { }\left\{ {Y{ } \in { }U/R:Y \subseteq X} \right\}$$

The basic goal of the rough set analysis is to infer (learn) approximations of concepts. It basically explains how to use arithmetic to find hidden patterns in data^128^. Data model information is maintained in a table in Rough Set. Each row represents a fact or an object (tuple). The facts often contradict each other. In Rough Set, a data table is referred to as an Information System. As a result, the information table can represent the input data for any domain. An information system is made up of a non-empty finite set of objects (U) and a non-empty finite set of attributes (A) (U, A). The elements of A are conditional characteristics. A decision table is defined as a table with one or more decision attributes. A decision system is defined as (U, A union d), where d is the decision attribute.

On tables, there are many things with similar qualities. One strategy for reducing table size is to store only one representative item for each set of objects with the same attributes. Indecipherable objects are often known as tuples. Any P subset A is related by an equivalence relation IND (P), where IND (P) stands for relation indiscernibility where x and y are indistinguishable from one another due to attribute P's nature. Indiscernibility is defined as follows in Eq. (17):

17 $$IND\left( P \right) = \{ \left( {x,y} \right) \in {\mathbb{U}}^{2} { }|{ }\forall_{a} \in P,a\left( x \right) = a\left( y \right)\}$$

**Figure 4:**
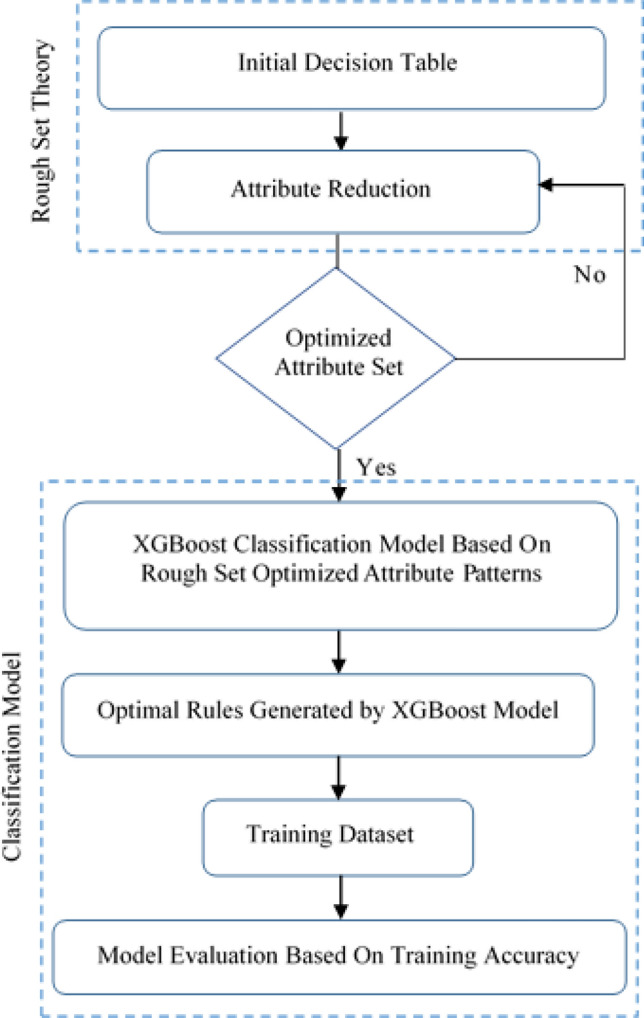
Fusion of rough set theory and XGBoost for landslide prediction modeling.

Following the successful discovery of hidden patterns, the next step is to feed these patterns into the landslide prediction model. Random Forest, Bagging, XGBoost, BPNN, and BN methods are used for classification and prediction. Random Forest, Bagging, XGBoost, BPNN, and BN classifiers receive the rules derived using the rough set technique as input for training purposes as shown in Fig. 4.

### Validation and comparison methods

A final model is not acceptable unless it is validated, as proven by landslide studies. To evaluate the landslide models in this work, the authors employed the receiver operating characteristic (ROC) and other statistical estimators such as accuracy and precision. The most widely used performance indicators were computed using the confusion matrix^127^ provided by the classifier. The entries in the confusion matrix have true positive (TP), false positive (FP), true negative (TN), and false negative (FN).

### Receiver operating characteristics (ROC) curve

ROC illustrates the percentages of true positive over false negative percentages to rank previous landslides cumulatively in decreasing order^129^. This includes determining success rates by utilizing the areas under ROC curves (AUROC). The AUROC is used to determine the prediction ability of a model. The AUROC values get smaller or equal to 50 when the prediction is poor, but AUC values closer to 100 indicate a reliable estimate. Furthermore, the greater the AUROC number, the better the model performance, and an AUROC value of 100 indicates exceptional performance. In recent studies, the AUROC curves have been recognized as one of the best and most often used tools for validating and comparing models. As a result, it has been widely used as a standard technique to analyze the model performance capability. The AUROC for the present study is computed using Eq. (18).

18 $$AUC = \left( {\frac{{{\Sigma }TP + {\Sigma }TN}}{P + N}} \right)$$

### Sensitivity, specificity, precision, accuracy, F1-score

Other than the ROC, popular and widely used statistical indices such as sensitivity or recall, specificity, precision, accuracy, and F1-score have also been used to assess the overall accurateness of the final results generated by various machine learning algorithms. Equations (19)–(23) establish the performance metrics under examination, for sensitivity, specificity, precision, accuracy, and F1 score. Sensitivity or recall is a metric that measures a model ability to predict real positive outcomes for each class label. In this study, sensitivity describes the right classification of landslide occurrence.

19 $$Sensitivity = \left( {\frac{TP}{{TP + FN}}} \right)$$

The proportion of genuine negatives correctly detected is determined by the specificity metric. When it comes to categorizing negative samples, a model with a high specificity performs better. It indicates how many real negative cases have been identified.

20 $$Specificity = \left( {\frac{TN}{{TN + FP}}} \right)$$

In comparison to all anticipated positive samples, the precision represents the fraction of genuine positive samples. The model high precision means that it has a good chance of successfully categorizing positive samples.

21 $$Precision = \left( {\frac{TP}{{TP + FP}}} \right)$$

The accuracy of a database is defined as the percentage of samples accurately predicted. Out of all potential classifications, this phrase represents how many right classifications were made. It denotes the proportion of “True” to the total number of "True" and "False".

22 $$Accuracy = \left( {\frac{TP + TN}{{P + N}}} \right)$$

F1 score is the harmonic mean of precision and recall with a maximum value of 1 and the minimum value of 0. Precision and Recall are weighted in the F1 score, meaning that FP and FN are equally relevant. This is a considerably more useful metric when compared to "Accuracy." The problem with utilizing accuracy is that if we train the model on a severely imbalanced dataset, the model will learn how to properly forecast the positive class but not how to identify the negative class.

23 $$F1{ }score = 2{*}\left( {Precision{*}Recall} \right)/\left( {Precision + Recall} \right)$$

## Results

### Independent test of causative factors

**Figure 5:**
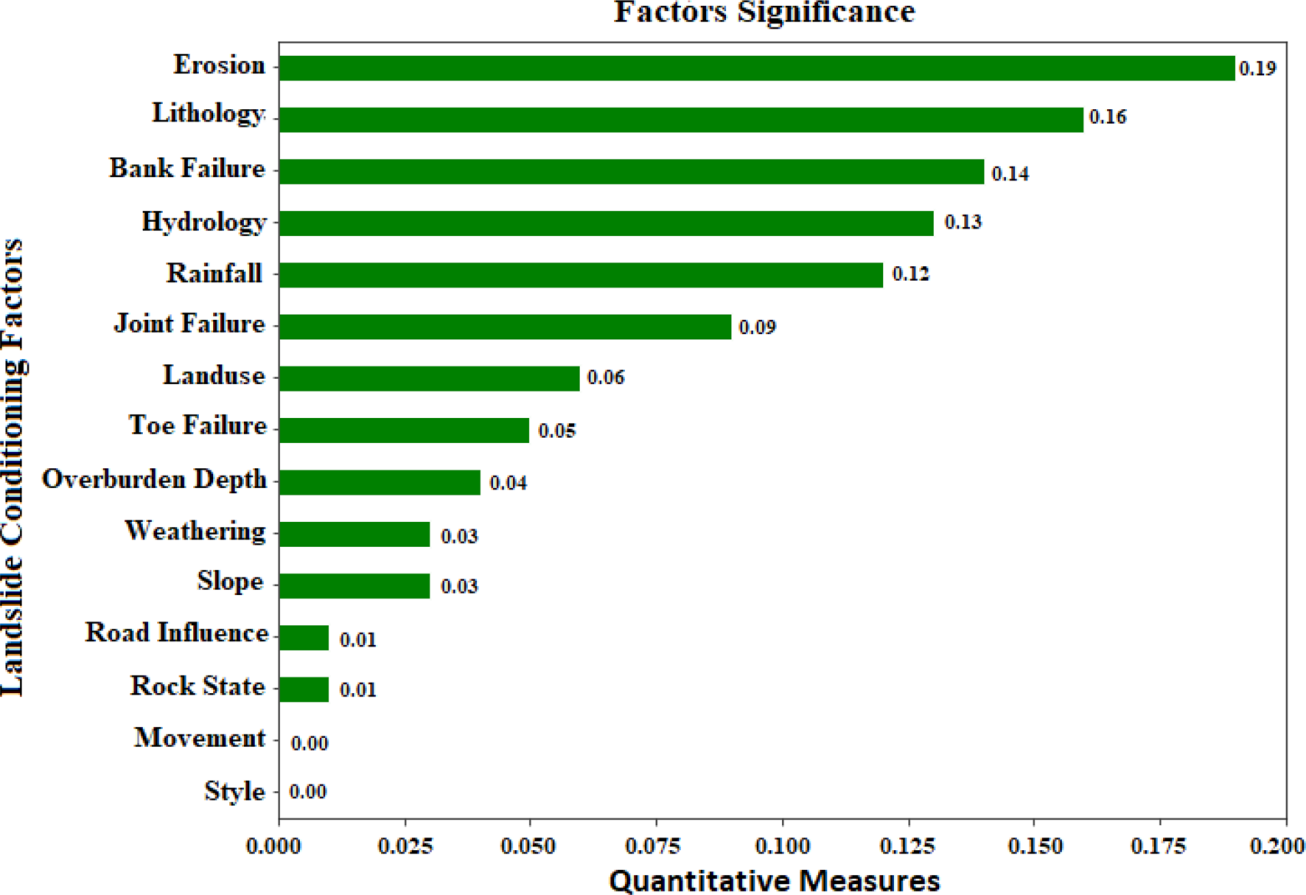
Significance of total 15 landslide conditioning factors using LASSO feature selection technique.

The authors used the LASSO technique to choose conditioning factors for the landslide prediction model for the Uttarkashi region. The factors were chosen based on their weights in order to fit with the landslide prediction models. For the landslide study, causes with weights greater than zero were evaluated. Factors with a weight below or equal to zero were, on the other hand, removed from landslide modeling. According to the calculated LASSO values listed in Table 2 and represented in Fig. 5, 13 out of 15 factors are significant in landslide prediction (Quantitative Measures > 0).

**Table 2:** Significance of landslide conditioning factors (LCFs) using LASSO technique.

Factor	Quantitative measures
Erosion	0.19
Lithology	0.16
Bank failure	0.14
Hydrology	0.13
Rainfall	0.12
Joint failure	0.09
Land use	0.06
Toe failure	0.05
Overburden depth	0.04
Weathering	0.03
Slope	0.03
Road influence	0.01
Rock State	0.01
Landslide movement	0.00
Landslide style	0.00

The results from Table 2 and Fig. 5, it is disclosed that erosion has the highest value of 0.19, followed by Lithology (0.16), Bank Failure (0.14), Hydrology (0.13), Rainfall (0.12), Joint Failure (0.09), Land use (0.06), Toe failure (0.05), Overburden Depth (0.04), Weathering (0.03), Slope (0.03), Road Influence (0.01), Rock State (0.01), Landslide Movement (0.00), and Landslide Style (0.00). However, because the two conditioning factors, landslide movement and landslide style, were assessed as null prediction capability (measure = 0), they have no positive relation. In order to increase the accuracy of the final output, both elements were not incorporated in the current landslide modeling.

### Multicollinearity test of causative factors

A good selection of predictive conditioning factors is required in this type of study to ensure that these conditioning are independent of one another. The linear relationship between distinct independent conditioning factors determines a models overall accuracy, and excessive multicollinearity might reduce the model's predictive capacity. Python programming was used to perform multicollinearity analysis on the selected dominating factors in this investigation. To identify the predictive conditioning factor and assess their multicollinearity, tolerance and VIF were utilized in this study. Table 3 reveals that the TOL value of all factors is greater than 0.1, and the VIF is less than 10, indicating that there are no severe multicollinearity issues. For this study, the factors with tolerance (≤ 0.1) and VIF (> 5) are eliminated. Therefore, the factors of landslide style and landslide movements are not considered for landslide prediction modeling.

**Table 3:** Multicollinearity analysis using tolerance and VIF for landslide conditioning factors used in landslide prediction modeling.

Factor	Tolerance	VIF
Bank failure	0.882102	1.133656
Joint failure	0.786793	1.270982
Erosion	0.736451	1.357864
Toe failure	0.665457	1.502726
Overburden depth	0.664323	1.505292
Road influence	0.436506	2.290920
Rainfall	0.387546	2.580337
Rock state	0.383740	2.605933
Lithology	0.360629	2.772933
Hydrology	0.332577	3.006826
Weathering	0.277825	3.599388
Slope	0.275084	3.635255
Land use	0.271750	3.679853
Landslide movement	0.144255	6.932168
Landslide style	0.103840	9.630224

**Figure 6:**
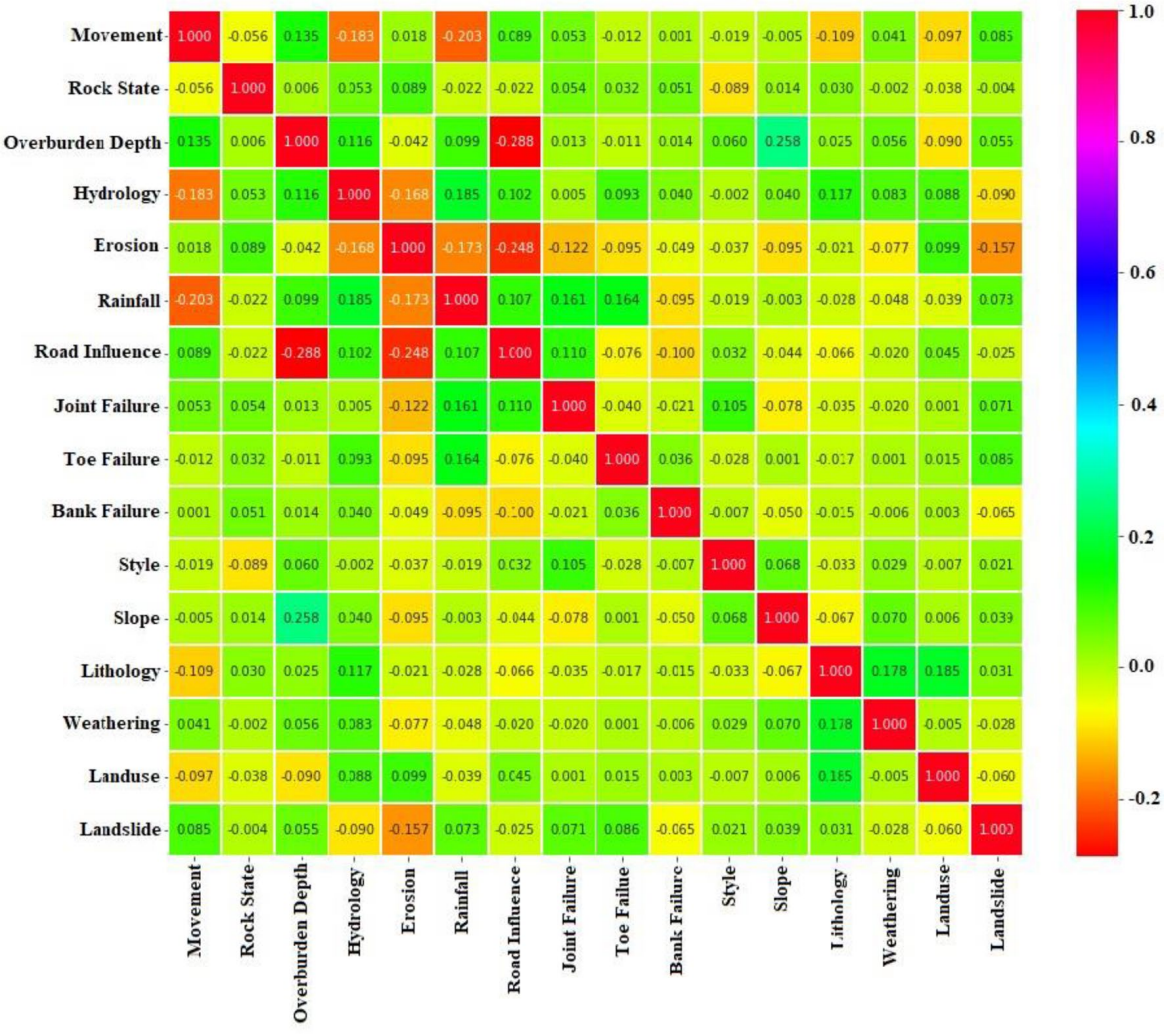
Heat map showing correlation matrix of landslide conditioning factors (LCFs). The color bar value of the right bar from high to low represent how closely the factors are correlated.

Additionally, a heat map is generated to visualize the correlation matrix between the conditioning factors shown in Fig. 6. The colour value of the right band that ranges from low to high [-0.2, 1] indicates how closely the factors are correlated.

### Model hyper-parameters tuning

The optimum hyperparameters for the ML models were discovered applying a grid search. The scikit-learn is used to implement all of the techniques in Python. HXGBRS and is suitable for building baseline models in landslide prediction analysis for contrastive ML models due to the fixed limit of parameters. For our proposed hybrid models, we trained and tested the model for a total of 554 instances. In the training process, different hyperparameters were tuned to improve the accuracy of the models. For HBNRS model, a grid search is performed tuning learning rate using values [0.1 to 0.9]. From the results, it was observed that = 0.1 was useful in yielding optimal accuracy. For HBPNNRS model, a grid search was performed to tune learning rate using values [0.01, 0.02, 0.03, 0.08, 0.09, 0.1, 0.2, 0.8, 1]. The learning rate at 0.2 produced the highest accuracy for the model. For HBRS, HXGBRS, and HRFRS models, a grid search was performed to tune a number of estimators from 10 to 1000. The HBRS model achieved the highest accuracy using 150 estimators, HXGBRS achieved the highest accuracy using 60 estimators and HRFRS achieved highest accuracy using 110 estimators.

### Models validation and comparison

**Figure 7:**
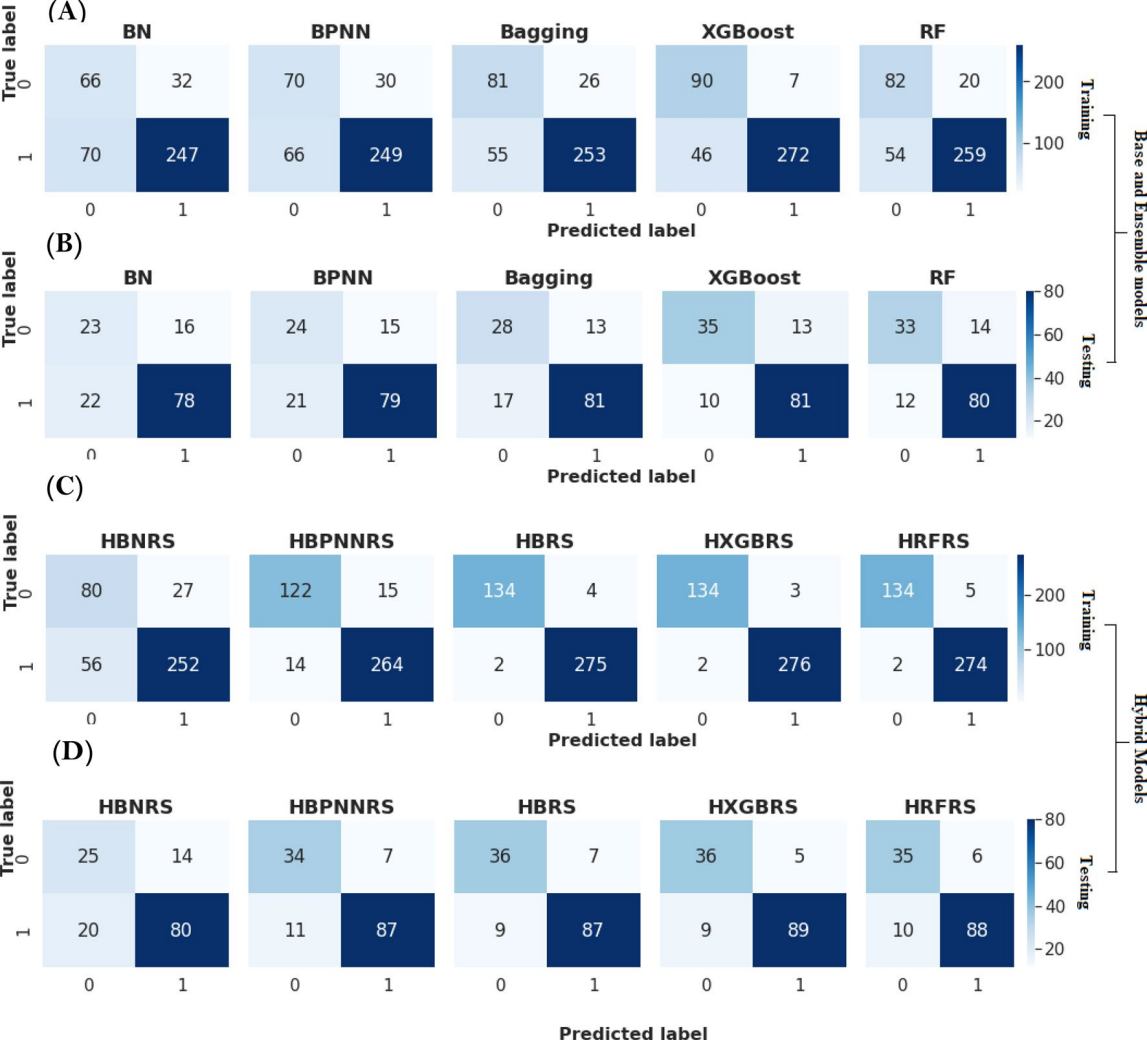
Confusion matrices showing classification results of landslides and non-landslides using base, ensemble and hybrid models based on training and testing dataset. It depicts the TN (top left), FP (top right), FN (bottom left) and TP (bottom right). (**A**) Represents the confusion matrix using base and ensemble models for training data. (**B**) Represents the confusion matrix using base and ensemble models for testing data. (**C**) Represents the confusion matrix using hybrid models for training data. (**D**) Represents the confusion matrix using hybrid models for testing data.

To improve the prediction capability of the landslide models, five hybrid ML approaches were implemented: Random Forest based Rough Set (HRFRS), Bagging based Rough Set (HBRS), XGBoost based Rough Set (HXGBRS), BPNN based Rough Set (HBPNNRS), and Bayesian Network based Rough Set (HBNRS). These hybrid models were constructed by combining the Rough set model with individual empirical methods. The rough set model is useful for identifying the optimized landslide patterns. These optimized patterns were utilized by individual empirical models for the training purpose of the model. The final model for landslide prediction in this work will be a hybrid method with the best prediction capabilities. The confusion matrices for the developed models using training and testing dataset is shown in Fig. 7.

**Figure 8:**
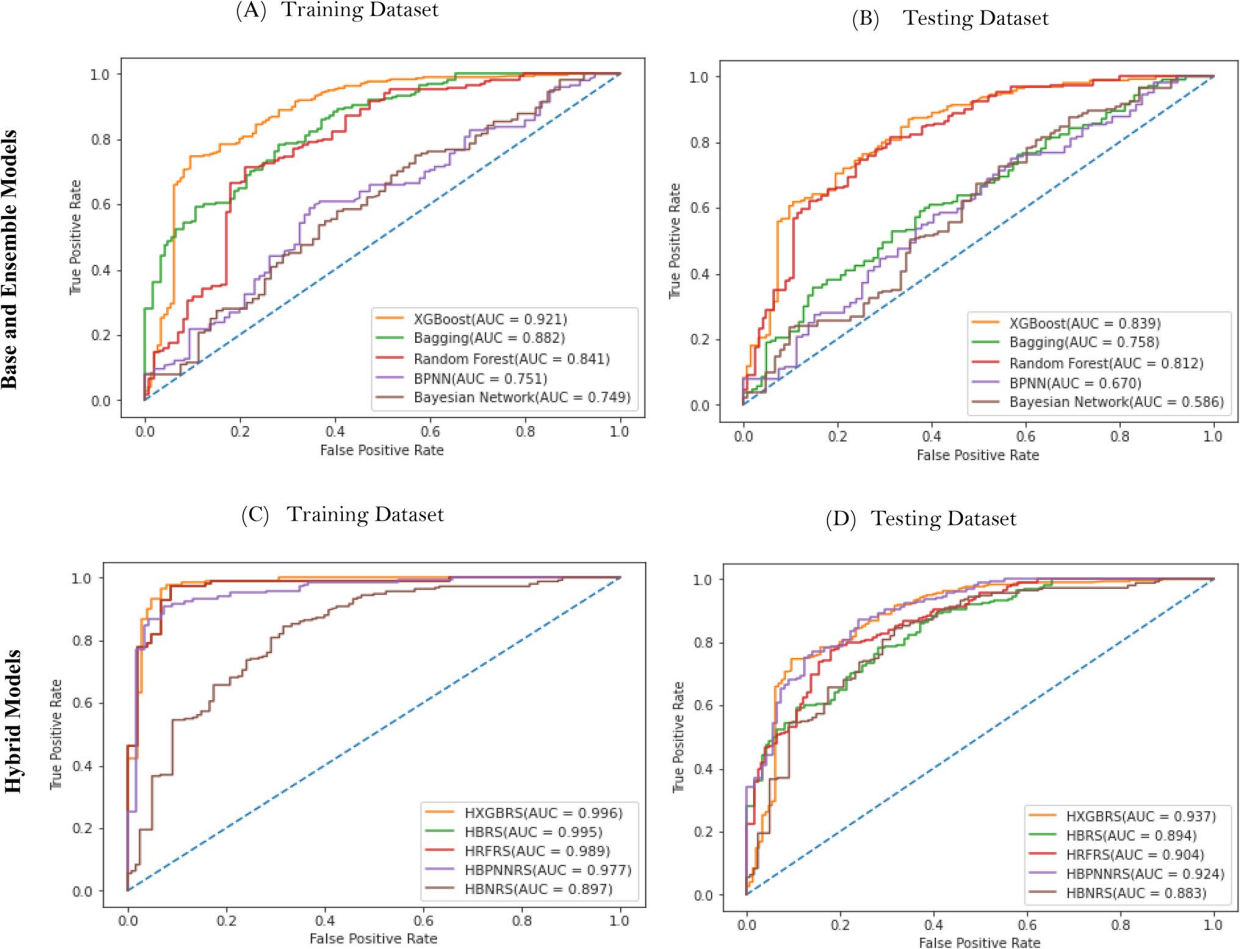
ROC curves used to analyze the prediction capability of various base, ensemble and hybrid landslide prediction models. (**A**) and (**C**) represents the performance for training dataset, (**B**) and (**D**) represents the performance for testing dataset.

The overall performance of the five approaches was evaluated using five statistical indicators (sensitivity, specificity, precision, accuracy, and F1-Score) and the AUROC curves as shown in Fig. 8. ROC curves have been employed in landslide hazard evaluation by a large number of researchers^130–133^^.^ The prediction accuracy of all models was measured using ROC in this study. The AUC of the ROC curve was calculated using training and testing datasets. The performance of the models showed acceptable outcomes as shown in Fig. 8. In both the training and testing datasets, the HXGBRS model obtained the highest AUC values. The AUC values for the HXGBRS model were determined to be (0.937), respectively, for the testing dataset followed by HBPNNRS (0.924), HRFRS (0.904), HBRS (0.894), and HBNRS (0.883) respectively listed in Table 5. All of the models, however, produced reasonable results and showed to be promising solutions for predictive modeling in the region under investigation.

Additionally, the models were compared and evaluated using five statistical estimators (accuracy, sensitivity, specificity, precision, and F1-score) listed in Tables 4 and 5. The results of the study revealed that the HXGBRS model had the highest accuracy, precision, and F1-Score values (89.92%, 0.946, and 0.926), followed by HRFRS (88.48%, 0.936, and 0.916), HBRS (88.48%, 0.925 and 0.915), HBPNNRS (87.00%, 0.905 and 0.895), and HBNRS (75.50%, 0.851 and 0.824) respectively. Furthermore, the HXGBRS model gained the highest specificity (0.878) followed by HRFRS (0.853), HBPNNRS (0.829), HBRS (0.837) and HBNRS (0.641) respectively. Finally, utilizing testing data, all of the landslide prediction models used in this study show an acceptable goodness of fit. Moreover, it is also observed that the individual predictive capability of the landslide models is improved when integrated with the rough set model listed in Tables 4 and 5.

**Table 4:** Performance comparison of various base, ensemble and hybrid landslide prediction models on training dataset using statistical parameters based on confusion matrix.

Performance Measures	Results on Training Dataset									
	BN	HBNRS	BPNN	HBPNNRS	RF	HRFRS	Bagging	HBRS	XGBoost	HXGBRS
Sensitivity	0.779	0.792	0.790	0.949	0.827	0.992	0.821	0.992	0.855	0.992
Specificity	0.673	0.747	0.700	0.890	0.803	0.964	0.757	0.971	0.927	0.927
Precision	0.885	0.903	0.892	0.946	0.928	0.982	0.906	0.985	0.974	0.989
F1-Score	0.861	0.850	0.837	0.947	0.874	0.986	0.861	0.988	0.881	0.990
AUC	0.749	0.897	0.751	0.977	0.841	0.989	0.882	0.995	0.921	0.996
Accuracy (%)	75.42	80.00	76.86	93.00	82.00	98.31	80.48	98.55	87.71	98.79

**Table 5:** Performance comparison of various base, ensemble and hybrid landslide prediction models on testing dataset using statistical parameters based on confusion matrix.

Performance Measures	Results on Testing Dataset									
	BN	HBNRS	BPNN	HBPNNRS	RF	HRFRS	Bagging	HBRS	XGBoost	HXGBRS
Sensitivity	0.780	0.800	0.790	0.887	0.869	0.897	0.826	0.906	0.890	0.908
Specificity	0.589	0.641	0.615	0.829	0.702	0.853	0.682	0.837	0.729	0.878
Precision	0.829	0.851	0.840	0.905	0.851	0.936	0.861	0.925	0.861	0.946
F1-Score	0.803	0.824	0.814	0.895	0.859	0.916	0.843	0.915	0.875	0.926
AUC	0.586	0.883	0.670	0.924	0.812	0.904	0.758	0.894	0.839	0.937
Accuracy (%)	72.66	75.50	74.00	87.00	81.00	88.48	78.41	88.48	83.45	89.92

### Dynamic visualization

The ability to visualize spatial data through dynamic mapping has enormous potential^134^. Multiple, short-term, significantly different views of a data set, each constructed with a specific query in mind, are a key component of the analytical process, and dynamic maps that display observer-related behaviour are especially ideal for data exploration^135^. This method allows for the enrichment of traditional cartography and statistical representations of spatial data with dynamic visuals and transient symbolism, which provide on-demand additional details about a symbol or statistic^134^. Maps that provide more than a single static view of a spatial data set, or those that change over time, are becoming more popular and easier to create^136^. Each of these observer-related strategies includes interacting with dynamic views of data by identifying entities in single or multiple views and using temporary symbolism to show some attribute of the entity in the graphics that depict them^137^. Map design for visualization may now be more concerned with establishing and providing an acceptable flexible framework for exploratory dynamic mapping than with appropriate representation^136^. The dynamic displays consist of an interactive interface customized to a certain set of users, which enables experimenting with different combinations of conditioning factors in landslide study^138^.

**Figure 9:**
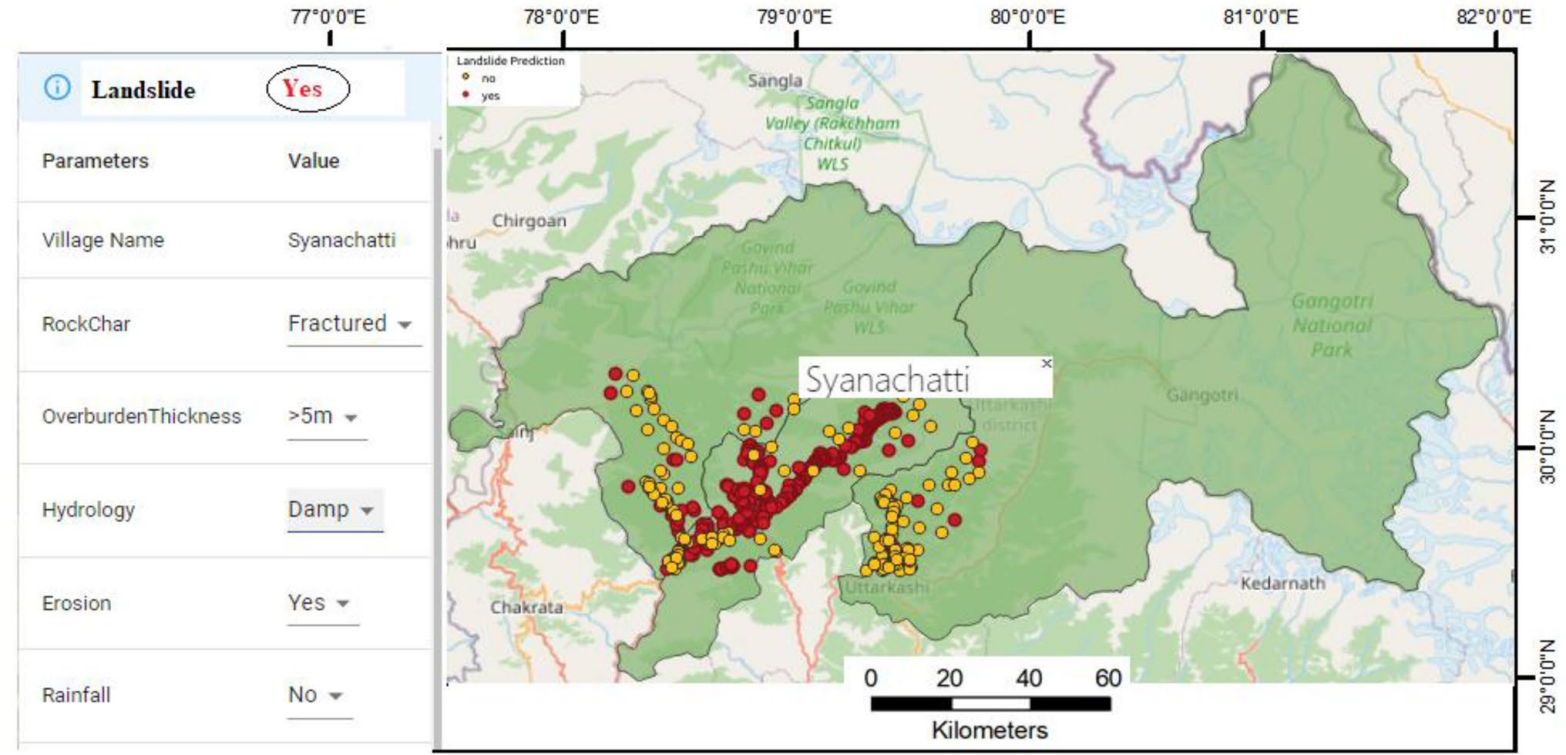
GIS based user interface to predict the possibility of landslide and non-landslide events on changing the values of landslide conditioning factors considered for the study area.

Visualizations are necessary for quickly identifying data issues that require more examination and analysis. For this study, a GIS-based user interface for dynamic interaction and visualization is built utilizing OpenStreet Map^139^ and study area shapefile. To begin, the research area landslide locations were merged into OpenStreet maps by using the PostgreSQL database management system to connect the map to the landslide inventory. Second, the landslide triggering patterns^140–142^ that had been identified using the HXGBRS method were used. The HXGBRS model provides the strongest prediction ability, according to the modeling findings. Thirteen landslide conditioning factors were considered in the current study to predict the landslide possibility in a non-landslide area. Landslides occur when each factor interacts with the others in some way. Users or analysts can adjust the values of the conditioning factors using the dropdowns option provided by the GIS-based user interface demonstrated in Fig. 9. The landslide class [yes, no] will vary if the user changes the value of any feature, according to the patterns detected. Analysts will be able to quickly detect the hidden combinations of elements that cause landslides with this approach.

## Discussion

Landslide prediction study is vital for land management, planning, and development in hilly mountainous locations^143^. This benefit makes this method a useful tool for improving the accuracy of predicting landslides dynamic geological and geomorphological conditions^68^. It's difficult to create a precise landslide prediction model. Many academicians throughout the world have employed a variety of approaches and procedures to develop realistic landslide prediction models, but the subject has prompted disagreement among researchers^12,27,30^. As a result, innovative methods for developing and implementing landslide prediction models must be created and applied. The discovery of innovative methodologies for landslide prediction analysis has resulted from ongoing research. The goal of this research is to compare hybrid landslide prediction models developed utilizing the rough set theory approach.

The parameters examined in this study have a major impact on landslide disasters. As a result, the relationship between the outcomes and the factor distribution must be examined. Although the role of LCFs varies by location, there is no question that a combination of geo-environmental elements acts as a landslide regulator^52^. Selecting the right landslide conditioning factors in landslide hazard modeling leads to more accurate results and less noise, improving the model's predictive abilities^144^. However, it is understood that there is no set procedure or standard for selecting LCFs^117^. As a result, in landslide modeling studies, selecting proper LCFs is considered an important task. In order to analyze the landslide vulnerability in Uttarkashi, Uttarakhand, fifteen LCFs were chosen as an independent factor. The LASSO model was used to quantify and analyse the involvement of LCFs in landslides in the research region. The crucial importance of the triggering component is perfectly captured by LASSO^116^. Erosional activities, which are the triggering condition used in this study, have the greatest LASSO value. The significance of land use factor demonstrates the landslide influence of anthropogenic activities. Because all landslide conditioning factors had LASSO values greater than zero, it may be concluded that all of the identified elements have continued to play a role in landslide occurrence. Landslides are caused by a complex collection of circumstances, and there is no single factor that causes them. Calculating multicollinearity is crucial for multivariable landslide modeling. Because the model overall precision is influenced by a clear correlation between the LCFs. To test for multicollinearity, among the independent conditioning factors, TOL and VIF were used. The results reveal that the identified landslide influencing factors are not multicollinear listed in Table 3. After the identification of the most significant influencing factors, the dataset was randomly split into training set (75%) and testing set (25%) for the 554 landslide and non-landslide locations Empirical studies frequently show that using 20–30% of the data for testing and the remaining 70–80% for training produces the best results^13,145–147^. To overcome the issue of underfitting and overfitting due to the size of the dataset, a series of runs with varied numbers of data and testing data [70:30, 75:30, and 80:20] were performed for this study. For this investigation, a 75:25 ratio was observed suitable. The accuracy obtained applying 75:25 split yield better results than 80:20 and 70:30 splits.

The main goal of the study is to create a reliable landslide prediction model that can forecast future landslides using regional conditioning factors. This would also aid in the identification of landslide-prone locations in the study region. For this study, five hybrid models namely HBNRS, HBPNNRS, HRFRS, HBRS, and HXGBRS models were trained for landslide prediction modeling. Basically, the predictive capability of two single models and three ensemble models was improved through rough set fusion. The advantage of using rough set techniques with single and ensemble models is rough sets can be used to generate optimized patterns of landslides that improve the prediction capability of constructed models. From the study, it is revealed that the HXGBRS model outperformed in comparison with other hybrid models.

The AUC of the ROC curve was used to validate and compare landslide models, as well as numerous statistical indicators (sensitivity, specificity, Precision, Accuracy, and F1-score) to reveal the models' prediction capacity. Both the training and testing datasets were taken into account while validating models. The findings reveal that all of the models performed well. HXGBRS has outperformed other hybrid models in terms of accuracy and prediction, as evidenced by the highest values of AUC and Precision (for both training and validation datasets) listed in Tables 4 and 5 and shown in Fig. 8. Additionally, the study revealed that the generated hybrid models and common single machine learning techniques were successfully contrasted with training and testing datasets. In the Uttarkashi district of Uttarakhand, hybrid XGBoost with a rough set has shown a higher landslide prediction capacity. However, the interpretation of the results of such methods requires considerable attention because the performance of hybrid models is determined by the models design, which includes the structure of the training data, and the size of the input data.

After comparing the findings of this study to those of previous studies, the following conclusions were reached. Models with similar performance do not however have the same prediction capabilities. The results of these hybrid machine learning methods differ from those of other research conducted in different parts of the world^84,148–150^ but they all indicate a high level of landslide prediction (AUC > 0.850). The fact that each expert used different dataset sources in his investigation illustrates the difference. The emergence of this variation in results was also influenced by differences in regional conditions^61^. As a result, the data selection and type, as well as the machine learning method, are critical.

Different modeling methodologies may produce different outcomes. The predicted performance of these hybrid machine learning models was shown to be more accurate than traditional statistical models for landslide prediction modeling in the majority of cases^64,86^. Because machine learning approaches are designed to automatically detect correlations between effective factors^151^. Comparing the results of the current study to those of earlier research using single or hybrid models, conducted in different, study regions and which had almost identical topographical and geological circumstances, it can be concluded that there is a difference in terms of the acquired AUC values and accuracy. In comparison to the approaches, namely Rotation Forest based Radial Basis Function (RFRBF) neural network (AUC = 0.891 and Accuracy = 82%)^152^, Bagging based Reduced Error Pruning Trees (BREPT) accuracy (AUC = 0.872 and Accuracy = 80%)^153^, and Support vector machine with cuckoo optimization algorithm(AUC = 0.738)^148^, the hybrid in the current study produced high performances for Himalayan regions in terms of AUC and overall accuracy AUC = 0.937 and Accuracy = 89.92%). Normally, because of the HXGBRS technique, the model can correctly detect the impact of specific predictors even when there is a lot of additive noise in the data. Moreover, the XGBoost ensemble alone is a powerful method that is capable of handling missing values and supports regularization^154^. Additionally, the rough set method helped in identifying the optimized landslide patterns that were later utilized by XGBoost classifier^128^.

Finally, a GIS based user interface is designed by the authors to predict the probability of landslides in the locations that are less or not probable of landslides. The reason behind this is the study area underlying geological characteristics are complex, with massive, sheared, fractured rocks occupying the majority of the region^88^. The rock structure is weak and unstable in shear. Because it is simple to vary due to atmospheric precipitation, surface water, groundwater, and land use, due to which the majority of slopes are expected to fall^92^. Human activities, particularly road development^94^, are primarily concentrated in the middle and low elevation range, which, when combined with slope toe excavation, accumulation, blasting, mining, and other activities, will increase the slope's instability. Around the same time, deforestation, vegetation transformation to cultivated land, and the building will create many open places, causing landslides to occur^155^. Therefore, the user interface Fig. 9 designed for this study will be helpful to the analysis to identify the hidden patterns that trigger landslides^134^. Landslide prediction modeling could be a useful visualization tool^135^ for preventing landslides, and enhancing the accuracy of the landslide prediction models is critical.

## Conclusion

Landslides have become an extremely sensitive concern in the Uttarkashi region in recent years. Landslides have been a nightmare for residents in this area due to relatively young, fresh, and fragile geological formations. During the study phase of this type of research, a detailed evaluation of the future likelihood of landslides is an essential way to proceed. As a result, demarcating possible landslide occurrence zones in steep terrain is critical for development, urban planning, and land management. A landslide prediction modeling can be considered a beneficial tool in this context. Machine learning approaches have already produced a highly accurate outcome and, as a result, have become well-known in recent years. For the examination of slope instability, different machine learning techniques were chosen. The main goal of this study is to compare the results of five hybrid machine learning techniques in order to determine which method is best for assessing landslide prediction in the study area. The AUC results show that all of the machine learning methods worked well. However, the XGBoost-based rough set outperformed other hybrid machine learning methods in terms of accuracy (89.92%) and predictive capability. Moreover, the prediction capability of individual models (BN, BPNN, RF, Bagging, and XGBoost) was improved using the optimized patterns generated by the rough set method. The results generated by the HXGBRS approach had the highest accuracy in our research; the reason for this could be that multiple types of single classifiers are utilized, increasing the diversity of the models, and the same type of base models may induce overfitting. The methodologies and predicting factors utilized in this study were chosen from data collected, the study's aims, and the environmental circumstances of the study area. Visualization has proven to be a beneficial tool for swiftly examining prediction outputs, making it a trustworthy tool for dealing with the landslide prediction model. Authors will continue to investigate the use of hybrid methods in the field of landslide research in the future, attempting to use more dynamic factors and optimized ML methods to improve model performance and provide powerful visualization techniques by including dynamic and detailed map layers useful for decision-makers and managers in landslide disaster prevention.

## Data Availability

The data used in this research is taken from the open-access platform of geological survey of India (GSI). This platform comprises publicly available reports based on field studies prepared by the Geological Survey of India (GSI, https://www.gsi.gov.in/webcenter/portal/OCBIS/pageReports/pageGsiReports?_adf.ctrl-state=1gq1usi84_5&_afrLoop=935857055668031#!).
